# The London School of Medicine for Women

**Published:** 1915-03-13

**Authors:** 


### The Hospital

The Workers' Newspaper of Administrative Medicine and Institutional Life, Administration, National Insurance and Health.

No. 1499, Vol. LVII. SATURDAY, MARCH 13, 1915. PRICE ONE PENNY.

### THE LONDON SCHOOL OF MEDICINE FOR WOMEN.

The interest in the medical education of women is by no means a purely restricted and particular one, and we are sure that many, other than those immediately concerned, will watch with good-will the efforts that are being made to improve the equipment and the accommodation of the London School. To put the matter at its lowest, there is the consideration that as women doctors exist, it is for the public welfare that they should be good doctors. Perchance in some vainglorious moment a misguided enthusiast may suggest that the designs of Nature have specially fitted women for the glorious ministry of medicine, but the leaders of the movement know perfectly well that only by discipline and training can their young friends be fitted to reach the measure of practical efficiency in their profession. Hence their appeal for funds to enlarge their school and to extend its laboratories. The appeal is for £25,000, of which a proportion has already been subscribed; and while the times are not too propitious for appeals other than those which take origin in the war, it is certain that money devoted to medical education may claim to serve a patriotic purpose. The life of the nation is not of to-day only, and foresight and faith must outrun the present national emergency.

The London School has some real and substantial achievements as witnesses to its worth. Out of the thousand or so women whose names are on the Medical Register it can claim to have educated some 600. It has secured and maintained a successful working arrangement with a general hospital where its students have been able to obtain their clinical and practical training. And, on the whole, it has cultivated a high aim in the education of its students, standing not for a mere minimum, but for something above this. There has been a real desire, so far as circumstances permit, to develop a true university spirit as distinct from the more narrow utilitarian rôle of the technical college. The influence of the school, too, has been steadily opposed to the notion that women practitioners can fairly, and with self-respect, practise medicine on the cheap, or that they can in any way depart from the best professional traditions. All these things are to the good as showing that the school is animated by a worthy spirit, and that its governing authorities have some practical gifts as well as high aims. In addition, there is the plain lesson of the record that the numbers of the school students are increasing, as showing both that there is a demand for women doctors and that there are candidates anxious to meet the demand. It is on professions of this order that the real claim of the school rests, and may fairly be pressed. In the very nature of things the School of Medicine for Women is a somewhat detached and sheltered institution, and is apt, perhaps, to lose the stimulating benefits of competition and outside criticism. This is all the more reason why it should emphasise its practical and robust side, and should, so far as possible, suppress the tearful sympathiser. There are occasions when this is not always easy, and when injudicious friends will overflow into sentiment and into the promise of the good time coming and the better order which the women doctors will bring. The wise advocate, we think, will be content to recite actual achievements, and will leave comparisons to be adjusted in the order of time.

The broad fact is that a number of women have found a life interest in medicine, and have shown their ability to practise it with advantage in one or other of its departments. Further, there are fields of missionary activity where medical women can render service and where no member of the opposite sex may enter. If other women, driven by necessity or attracted by taste, desire a similar career, why should opportunity be denied them? The test as to the wisdom of their choice time will provide, and it will be in this manner, and not by any fashionable enthusiasms, that the extent of the movement will be determined. "I was ever of opinion," says Goldsmith's vicar, "that the honest man who married and brought up a large family, did more service than he who continued single and only talked of population." Yet there are some who cherish a contrary view, or who are denied the active opportunity, and this being so, why should they be hindered in their choice? Indeed, the question has been settled already in their favour, and the London School of Medicine for Women has earned the support for which it now appeals. In fact, it is somewhat extraordinary that the school has not already attracted greater support, or found a wealthy benefactor to make it the chief object of his public enterprise. The old days of opposition and prejudice are virtually dead, and a "cause" like that of the London School should not go begging in a day when "causes" less good invite and obtain endowment.

